# SAXS Studies of the Endoglucanase Cel12A from *Gloeophyllum trabeum* Show Its Monomeric Structure and Reveal the Influence of Temperature on the Structural Stability of the Enzyme

**DOI:** 10.3390/ma7075202

**Published:** 2014-07-17

**Authors:** Lis S. Miotto, Caio V. dos Reis, Mario de Oliveira Neto, Igor Polikarpov

**Affiliations:** 1 Grupo de Biotecnologia Molecular, Instituto de Física de São Carlos, Universidade de São Paulo, Av. Trabalhador São-Carlense 400, Arnold Schimidt, São Carlos, SP 13566-590, Brazil; E-Mails: lismiotto@yahoo.com.br (L.S.M.); caio.reis@usp.br (C.V.R.); 2 Departamento de Física e Biofísica, Instituto de Biociências de Botucatu, Universidade Estadual Paulista, Distrito de Rubião Júnior, S/N, Botucatu, SP 18618-970, Brazil; E-Mail: mario.neto@ibb.unesp.br

**Keywords:** endoglucanase, *Gloeophyllum trabeum*, biophysics, circular dichroism, small angle X-ray scattering (SAXS), Kratky analysis

## Abstract

Endoglucanases are key enzymes applied to the conversion of biomass aiming for second generation biofuel production. In the present study we obtained the small angle X-ray scattering (SAXS) structure of the *G. trabeum* *endo*-1,4-β-glucanase Cel12A and investigated the influence of an important parameter, temperature, on both secondary and tertiary structure of the enzyme and its activity. The CD analysis for GtCel12A revealed that changes in the CD spectra starts at 55 °C and the *T*_m_ calculated from the experimental CD sigmoid curve using the Boltzmann function was 60.2 ± 0.6 °C. SAXS data showed that GtCel12A forms monomers in solution and has an elongated form with a maximum diameter of 60 ± 5 Å and a gyration radius of 19.4 ± 0.1 Å as calculated from the distance distribution function. Kratky analysis revealed that 60 °C is the critical temperature above which we observed clear indications of denaturation. Our results showed the influence of temperature on the stability and activity of enzymes and revealed novel structural features of GtCel12A.

## 1. Introduction

Brown rot fungi are the most important agents involved in the degradation of wood products, which occurs both by enzymatic and non-enzymatic mechanisms. During this process, these fungi actively metabolize the carbohydrate portion of wood by breaking down cellulose and hemicellulose, leaving the lignin portion intact [1]. This feature holds an interesting property for industrial bioconversion [2].

*Gloeophyllum trabeum* is a representative specimen of this group and causes a typical brown rot, following peculiar pattern of wood decay during which wood cell wall components are oxidatively broken down, causing a rapid loss of wood strength [1]. The depolymerization of cellulose and hemicelluloses from the plant cell wall by *G. trabeum* occurs through the generation of hydroxyl radicals via the Fenton reaction (H_2_O_2_ + Fe^2+^) assisted by hydroquinones, and secretion of low molecular weight (*M*_w_) peptides. The hydroxyl radicals in turn become able to depolymerize plant cell wall polysaccharides due to their strong oxidizing capability [3].

Endoglucanases (EGs) also known as *endo*-1-4-β-glucanases are a type of cellulase that attack initially and randomly multiple internal sites of the amorphous regions of the cellulose fiber. These provide sites for subsequent attack by cellobiohydrolases [4]. EGs catalyze the hydrolysis of internal connections, β-1,4-d-glucosidic linkages in cellulose, and are also capable of hydrolyzing β-1,4-d-glucans also containing β-1,3 bonds [5,6]. EGs and other glycoside hydrolases and transglucosidases have been classified into glycoside hydrolases (GHs) families according to their substrate specificity and molecular mechanism in a Carbohydrate-Active EnZymes (CAZy) database [7,8].

The glycoside hydrolase family 12 in which GtCel12A is included can be divided into subfamilies and very few examples of GH12 family members have carbohydrate-binding modules (CBMs). GH12 members are found in a wide range of organisms including numerous extremophile species such as the archeon *Pyrococcus furiosus* [9], the hyperthermophilic bacteria *Thermotoga neapolitana* [10],the thermophilic eubacteria *Rhodothermus marinus* [11] and the thermotolerant acidophilic bacteria *Acidothermus cellulolyticus* [12].

In this study, we determined the small angle X-ray scattering (SAXS) structure and investigated the effect of temperature on the structural stability, compactness and activity of the *endo*-1,4-β-glucanase Cel12A from *G. trabeum*, a GH12 member, following different biophysical and biochemical approaches.

## 2. Results and Discussion

### 2.1. GtCel12A Is One of the Most Thermal Stable among Other Characterized Cel12A

Analysis of GtCel12A by circular dichroism (CD) revealed a predominant β-strand structure, which corroborated the typical β-sheets based fold commonly found in GH12 family members. Deconvolution of the GtCel12A spectrum showed that its secondary structure consists of 35.1% β-strands, 19.3% β-turns, 6.9% helices and 38.7% disordered residues.

**Figure 1:**
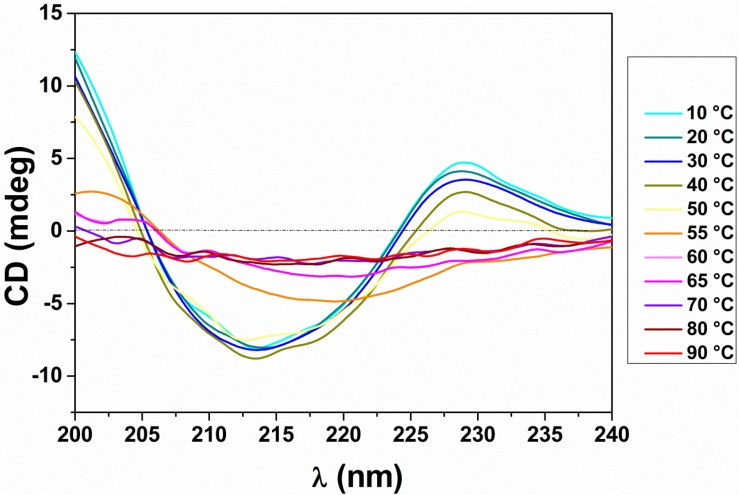
Circular dichroism (CD) spectra for the thermal denaturation assay of GtCel12A. The temperature range is from 10 to 90 °C varied in steps of 10 °C in the beginning of the experiment and steps of 5 °C above 50 °C.

Thermal denaturation assay revealed that changes in the CD spectra were observed above 55 °C, suggesting a perturbation in the secondary structure for GtCel12A (Figure 1). Boltzmann fit to the experimental curve on pH 3.0 revealed an apparent thermal midpoint (*T*_m_) of 60.2 ± 0.6 °C. This value is slightly higher than the *T*_m_ values previously reported for other Cel12A from different species, also determined by CD analysis. The Cel12A from *Trichoderma reesei* showed a *T*_m_ of 54.4 °C, 49.2 °C was observed for the Cel12A from *Hypocrea schweinitzii*, 58.5 °C for *Trichoderma koningii*, 54.1 °C for *Fusarium javanicum* and 45.9 °C for *Gliocladium roseum.* Only the Cel12A from *Streptomyces* sp.showed a higher *T*_m_ of 65.7 °C [13]. Moreover, the *T*_m_ determined by CD analysis agrees with the *T*_m_ equal to 60.4 ± 0.6 °C determined for this enzyme at the same pH using Thermofluor method in our previous experiments (data not shown).

### 2.2. GtCel12A Forms Monomers in Solution

The native polyacrylamide gel electrophoresis (native PAGE) results showed a single band, suggesting sample homogeneity and only one oligomeric form for GtCel12A, which was found suitable for further SAXS analysis.

**Figure 2:**
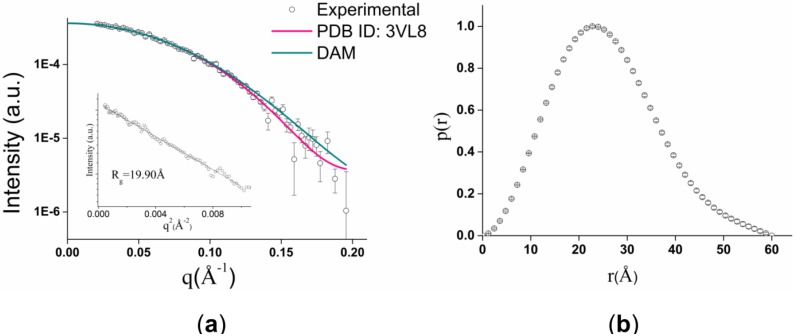
GtCel12A analysis by small angle X-ray scattering (SAXS). (**a**) Experimental scattering curve (open circles) and fits produced both from the homologous xyloglucan-specific *endo*-beta-1,4-glucanase from *Aspergillus aculeatus* (AaXEG) structure (PDB ID: 3VL8) (pink line) and from dummy atom model (DAM) (green line). The insert contains Guinier profile to calculate *R_g_*; (**b**) p(r) computed from experimental data.

Aiming to obtain information about size, molecular shape and oligomeric state of the GtCel12A in solution, we submitted the protein to SAXS analysis. Comparative analysis of SAXS scattering curves showed that concentration effects were negligible. With the aim of removing small aggregation effect, the *q*_min_ value was reduced to 0.02 Å^−1^. GtCel12A SAXS curve at a concentration of 1 mg·mL^−1^ is shown in Figure 2a.

The Guinier analysis, in the *q*^2^-range *R*_g_*.q* < 1.3, reproducibly gave estimates of 19.9 Å for the enzyme gyration radius (*R*_g_). The linearity of the Guinier plot indicated that the preparation was monodisperse.

Analysis of the distance distribution function [p(r)] led us to conclude that the protein had an elongated form with a maximum diameter (*D*_max_) of 60 ± 5 Å (Figure 2b). The *R*_g_ value of 19.4 ± 0.1 Å calculated from the p(r) corroborates with the Guinier analysis derived estimate.

The protein *M*_W_ estimate of 28.3 kDa was obtained using SAXS MoW web tool [14]. Considering an average 10% error of the *M*_w_ estimates obtained using SAXS MoW [14], this result agrees well with the *M*_w_ of GtCel12A monomer computed from its primary sequence (26.1 kDa) by EXPASy Prot Param web tool [15].

**Figure 3:**
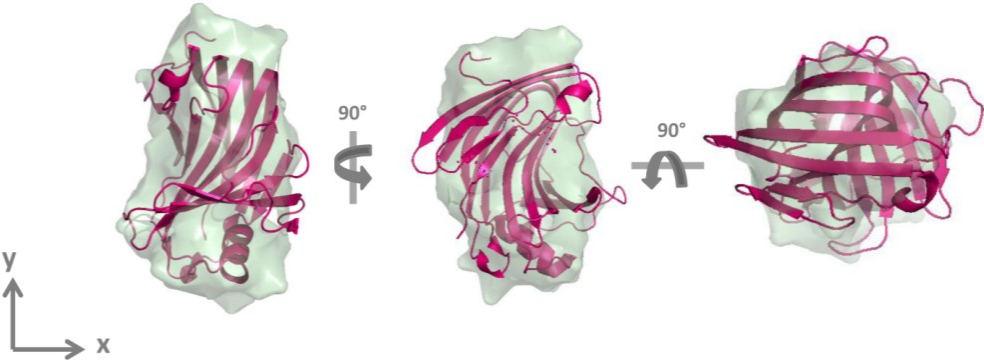
Structural superposition of the crystallographic structure of the homologous AaXEG (pink cartoon) and averaged DAM obtained for GtCel12A by SAXS analysis (green) shown in three different orientations. Right and center models are rotated 90° around *y* and *x* axis, respectively from the left model.

The DAM for GtCel12A was determined from SAXS data using Gasbor program [16]. Ten independently generated DAMs fitted the experimental SAXS data well. The superposition of DAMs based on normalized spatial discrepancy (NSD) parameter [17] with the homologous AaXEG [18] is shown in Figure 3.

The structural parameters derived from the experimental curve, from the homologous structure and from DAM and the fitting adjustment parameter χ are shown in Table 1. The values obtained from these different calculations are similar. 

**Table 1:** GtCel12A and homologous AaXEG structural parameters.

Parameters	Experimental	PDB ID: 3VL8 (AaXEG)	DAM
*R*_g_ (Å)	19.4 ± 0.1	18.25	18.57
*D*_max_ (Å)	60.00	53.09	56.78
Resolution (Å)	32.14	–	32.14
SAXS *M*_W_ (kDa) (theoretical *M*_W_ = 26.17)	28.30	–	–
χ	–	1.40	1.47

Detection of conformational switching, destabilization or long-range delocalized flexibility in solution can be investigated by SAXS, typically through qualitative assessments of the scattering data in a Kratky Plot [19,20].

**Figure 4:**
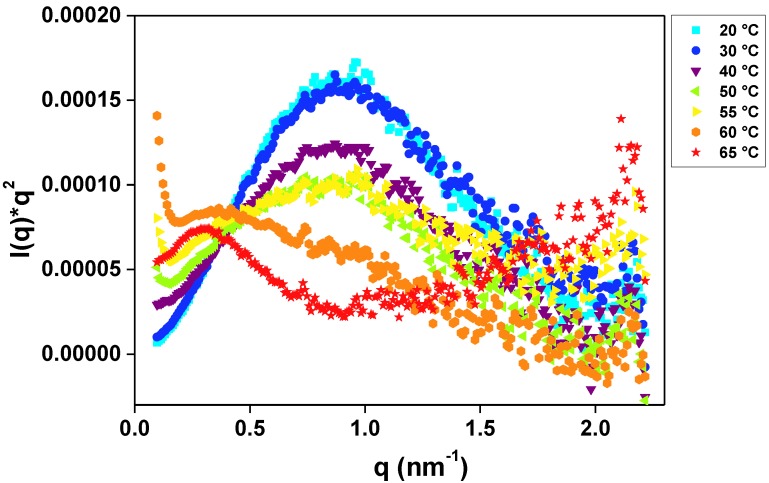
Kratky profile [*I* (*q*)*q^2^ × *q*] for GtCel12A in 50 mM sodium citrate buffer, pH 3.0 and temperature range from 20 to 65 °C. The presence of a parabolic peak for each temperature reports on the maintenance of enzyme globularity. The progressive peak decrease with no change in horizontal position indicates the loss of tertiary folding. At 60 °C, in addition to flattening of the peak, one can observe a shift to the left in its position. This suggests partial loss of globularity and possible aggregation of the enzyme molecules in solution with simultaneous increase in the *M*_w_ of the new ensemble.

Kratky plot is represented by [*I*(*q*)*q^2^ × *q*] curve [20,21], as shown in Figure 4. For small *q* values and globular enzymes, a parabolic peak is expected to be observed [20,21]. Considering high *q* values, molecule flexibility can be analyzed: the more ascending the end of the curve is, the more flexible the molecule is [20,21]. The *R_g_* and the *M*_w_ of GtCel12A remained unaltered up to 55 °C. From 60 °C on, the parabolic peak shifted to the left, representing an increase in both *R_g_* and *M*_w_, followed by the abrupt flexibility increase at 65 °C. Therefore, the peak shift indicated that the enzymes in solution were rearranged in a different molecular shape.

In the independent set of measurements we determined enzymatic activity of GtCel12A using β-glucan from barley at 1% as a substrate (to be published elsewhere) which showed that the enzyme’s activity began to decrease gradually above 53 °C and a sudden drop in activity was observed at a critical temperature of 60 °C. At higher temperatures, negligible enzymatic activity was observed, suggesting that most of the molecules were denatured. This result corroborates with SAXS analysis, which showed, by analyzing the molecular shape of the enzyme, that at 60 °C a clear thermal denaturation occurs, therefore affecting, both the structure stability and activity of GtCel12A.

## 3. Experimental Section

### 3.1. Gene Cloning, Recombinant Expression, Protein Production, Purification and Identity Confirmation

Complementary DNA was prepared by reverse transcription of total RNA isolated from *G. trabeum* strain ATCC 11539 grown in a medium containing barley and alfalfa 1% (w/v) for 32 h at 34 °C. The gene sequence was selected from GenBank (acession number HQ163778).

Gene cloning into vector ANIp7G, a vector previously constructed from ANIp7 [22] was carried out using the ligation-independent cloning (LIC) method previously described [23]. Recombinant DNA was transformed into *Aspergillus niger* py11 (*cspA*^−^ *pyrG*^−^ *∆Gla::hiG*), derived from strain N593 (*cspA*^−^ *pyrG*^−^), using the protoplast transformation method previously described [24]. Transformants were selected on minimal medium (MM) [25] without uracil and uridine. The ANIp7G vector without the inserted DNA was included as control. Protein production was performed in MM J media, which is similar to MM except that it includes 4% (w/v) of maltose as the sole carbon source and the amounts of nitrogen source, salts and trace elements were increased 4-fold. GtCel12A purification was performed in two steps using SP Sepharose, an ion-exchange column, as the first purification step. Fractions were eluted with a linear gradient of 0–1 M of sodium chloride in 50 mM sodium citrate buffer (pH 3.0) at a flow rate of 1 mL·min^−1^. Samples were analyzed by sodium dodecyl sulfate polyacrylamide gel electrophoresis (SDS-PAGE) and the fractions containing the protein of interest were combined and concentrated using a 10 kDa cut-off Vivaspin™ (GE Healthcare, Little Chalfont, UK) concentrator. In a second purification step, the concentrated volume was applied to a Superdex™ 75 10/30 column (GE Healthcare, Little Chalfont, UK) equilibrated with the same buffer complemented with 0.2 M of sodium chloride. 

Protein identity was further confirmed by liquid chromatography-mass spectrometry (LC/MS) analysis using MicroTOF-QII (Bruker Daltonics, Billerica, MA, USA) equipment after trypsin gel digestion of the band representing GtCel12A.

### 3.2. CD

Aiming to determine the secondary structure content of GtCel12A, samples of the purified protein at a concentration of 150 µg·mL^−1^ in 50 mM sodium citrate buffer pH 3.0 were placed in a quartz cuvette of 0.1 cm path length for CD measurements at 10 °C.

The GtCel12A CD spectra were assessed by far-UV CD spectroscopy using a J-815 Jasco spectropolarimeter equipped with a Jasco Peltier PTC 423S/15 (JASCO, Tokyo, Japan) temperature control unit. The CD spectra were collected using a wavelength range of 260–195 nm with a scanning speed of 100 nm·min^−1^, a spectral bandwidth of 1 nm and a response time of 0.5 millisecond. The protein signal was obtained by subtracting buffer spectrum from the sample spectrum and represented the average of 8 accumulations. The CD spectra deconvolution was performed with the program CONTINLL from the package CDPro [26].

A thermal denaturation assay was also performed by measuring the ellipticity changes induced by a temperature increase from 10 to 90 °C, in steps of 10 °C in the beginning of the experiment and steps of 5 °C above 50 °C and wavelength ranging from 240 to 200 nm. The apparent *T*_m_ was further calculated from the experimental CD sigmoid curve using the Boltzmann function.

### 3.3. Native PAGE

Protein samples of GtCel12A in 50 mM sodium citrate buffer, pH 3.0 in two different concentrations, 0.5 and 1.0 mg·mL^−1^ were analyzed by native PAGE. The experiment was performed in the equipment Phast System (Amersham Biosciences, Little Chalfont, UK), using a nondenaturing gel, the Phast Gel 8-25 with acrylamide gradient of 8%–25% (GE, Little Chalfont, UK). Proteins with high *M*_w_s were used as markers, such as bovine thyroglobulin (669 kDa), horse ferritin (445 kDa), catalase (232 kDa), aldolase (140 kDa) and bovine serum albumin (66 kDa). The gel was stained with Coomassie Blue in an aqueous solution of 25% methanol and 5% acetic acid.

### 3.4. SAXS Studies

SAXS data for GtCel12A at concentrations of 1 and 6 mg·mL^−1^ were collected on the SAXS2 beamline at the Brazilian Synchrotron Light Laboratory (LNLS/CNPEM-ABTLuS, Campinas, Brazil). The radiation wavelength was set to 1.48 Å and a 165 mm MAR-165 CCD detector (Rayonix, Evanston, IL, USA) was used to record the scattering patterns. The sample-to-detector distance was set to 1474 mm to give a scattering vector-range from 0.01 to 0.22 Å^−1^, where *q* is the magnitude of the *q*-vector defined by *q* = 4πsinθ/λ (2θ is the scattering angle). Protein samples were prepared in 50 mM sodium citrate buffer pH 3.0. The integration of SAXS patterns were performed using Fit2D software [27] and the curves were scaled by protein concentration. *R_g_* was approximated using two independent procedures: by Guinier equation [28] and by indirect Fourier transform method using GNOM program [29]. The p(r) was also evaluated by GNOM and *D*_max_ was obtained. Protein *M*_w_ was calculated using the procedure implemented on SAXSmoW web tool [14]. DAMs were calculated from the experimental SAXS data using ab initio procedure implemented in Gasbor program [16]. Several runs of ab initio shape determination with different starting conditions led to self-consistent results as judged by the structural similarity of the output models, yielding nearly identical scattering patterns and fitting statistics. CRYSOL program [30] was used to calculate the simulated scattering curve from the homologous AaXEG [18], a GH12 member, which showed 44% of sequence identity with GtCel12A. The evaluation of *R*_g_ and *D*_max_ was also performed with the same program.

Finally, the global compactness of GtCel12A in pH 3.0 was analyzed in terms of the Kratky plot [20], using a thermal variation assay with temperatures ranging from 20 to 65 °C in steps of 10 °C in the beginning of the experiment and steps of 5 °C above 50 °C.

## 4. Conclusions

The search for enzymes with improved activity and stability has been the goal of many studies in the context of enzymatic hydrolysis of biomass. Such improvement of enzymes, as well as the discovery of new enzymes, is key to reducing the costs of converting cellulose to ethanol. In this study, we obtained important data about the structure of GtCel12A in solution, such as its oligomeric state, dimensions, shape and information about molecule flexibility. The influence of temperature was investigated and confirmed that this is an important parameter to be considered for the structural stability of GtCel12A and other enzymes applied to second generation biofuel production.
